# Within‐ and between‐person relationships between spontaneous self‐affirmations, coping style, and wellbeing

**DOI:** 10.1002/smi.3145

**Published:** 2022-03-30

**Authors:** Taylor‐Jane Sharouni, Rachel G. McClymont, Christopher Alcorn, Amanda L. Rebar, Kwok Hong Law, Ben Jackson, Nerina Caltabiano, James A. Dimmock

**Affiliations:** ^1^ Department of Psychology James Cook University Townsville Queensland Australia; ^2^ Motivation of Health Behaviours Lab Appleton Institute School of Health, Medical and Applied Sciences Central Queensland University Rockhampton Queensland Australia; ^3^ School of Human Sciences (Exercise and Sports Science) University of Western Australia Perth Western Australia Australia; ^4^ Telethon Kids Institute Perth Children's Hospital Nedlands Western Australia Australia

**Keywords:** affect, approach coping, eudaimonic, hedonic, self‐affirmation, spontaneous, wellbeing

## Abstract

Self‐affirmations—responding to self‐threatening information by reflecting on positive values or strengths—help to realign working self‐concept and may support adaptive coping and wellbeing. Little research has been undertaken on spontaneous self‐affirmations in response to everyday threats, and less has been undertaken on the relationships between spontaneous self‐affirmations, coping, and wellbeing. This study aimed to test both within‐ and between‐person relationships between spontaneous self‐affirmations, coping, and wellbeing, controlling for threat intensity and other outcomes. A repeated survey assessment design was adopted to achieve these aims. Outcome measures included approach coping, avoidance coping, positive affect, negative affect, and eudaimonic wellbeing. It was found that spontaneous self‐affirmations positively predicted approach coping and positive affect at both within‐ and between‐person levels, and eudaimonic wellbeing at the between‐person level. Overall, spontaneous self‐affirmations were positively associated with approach coping and aspects of wellbeing.

## 1 INTRODUCTION

People often face stressors that can threaten the perception of themselves as adaptively and morally adequate (i.e., self‐integrity; Steele, 1988). The pervasiveness of these stressors is such that for many people, challenges to values, beliefs, relationships, and perceptions of competence are daily occurrences. Such exposure to self‐threatening information can undermine one's self‐concept and self‐esteem, and over time, can cause a host of other detrimental psychological outcomes (Dickerson, Gable, et al., 2009). In addition to their negative psychological consequences, self‐threatening stressors have been linked to increased cortisol release and proinflammatory cytokine activity (e.g., Dickerson, Gable, et al., 2009; Dickerson, Gruenwalkd, et al., 2009), as well as other negative physical health outcomes (e.g., mild cognitive impairment; Flier et al., 1998). Responding successfully to self‐threatening information is paramount for the maintenance of psychological and physical health, and individuals can employ several coping strategies in response to these challenges (Sherman & Cohen, 2006). First, individuals can accept threat and problem‐solve threatening information or events (Carver et al., 1989; Folkman & Lazarus, 1980; Sherman & Cohen, 2006). This process may be difficult to adopt, however, especially if issues threaten important domains related to an individual's self‐identity (Sherman & Cohen, 2006). An alternative coping response is to psychologically adapt to threat, such as by using distraction techniques or dismissing the importance of issues (Blanton et al., 1997; Carver et al., 1989; Folkman & Lazarus, 1980; Sivanthan et al., 2008; Wakslak & Trope, 2009).

A third alternative to coping with self‐threatening information—which may precede adaptive problem solving in relation to threat—is to affirm the self. Self‐affirmations are acts that demonstrate a person's adequacy; they typically involve reflections on, or engagement in activities related to, a strength or value held by the person (Sherman & Cohen, 2006). There have been many types of experimental inductions of self‐affirmation, although most of these inductions invite participants to focus on values or positive characteristics that are separate to the domain under threat (see McQueen & Klein, 2006). By attending to positive aspects of the self when a separate self‐domain is challenged, individuals are presumed to broaden their working self‐concept and uphold a global sense of self‐integrity (Cohen & Sherman, 2014; Critcher & Dunning, 2015; Sherman & Cohen, 2006). Self‐affirmations have been shown to be related to two high‐order, or umbrella, concepts of wellbeing—hedonia and eudaimonia (Emanuel et al., 2018; Nelson et al., 2014). Broadly, hedonic wellbeing involves experiences of pleasure, enjoyment, and comfort; hedonic ends include both physical and emotional‐cognitive pleasures and comforts (Huta & Ryan, 2010). Eudaimonia is more broadly defined, but generally reflects a pursuit of using and developing the best in oneself, in line with one's deeper principles (Huta & Ryan, 2010). Hedonia and eudaimonia are considered as complementary psychological functions (Huta, 2015)—it is possible, for example, that experiences of eudaimonia promote aspects of hedonia (such as positive affect; see Ryan et al., 2008).

With respect to hedonic wellbeing, self‐affirmations have been linked with improved positive affect and/or reduced negative affect in both experimental and large‐scale cross‐sectional work (e.g., Emanuel et al., 2018; Nelson et al., 2014). In a study involving over 3000 participants, for example, Emanuel et al. (2018) found that self‐affirmations were associated with greater happiness and hopefulness, and less sadness and anger. In relation to eudaimonic wellbeing, Nelson et al. (2014) found that experimentally‐induced written self‐affirmations were predictive of need satisfaction, meaning, and flow across multiple weeks. These effects were found in studies undertaken in both South Korea, in which participants were invited to affirm their values for 2 weeks, and the USA, in which participants undertook the values affirmation task for 4 weeks (Nelson et al., 2014).

Sherman and Cohen (2006) have suggested that self‐affirmations work to protect wellbeing, at least in part, due to their potential to reduce defensive processes. Because self‐affirmations reinforce the global self as ‘adaptively and morally adequate’ (Steele, 1988, p. 262), self‐affirmed individuals experience less need for restoration of self‐integrity via defensive processes such as disengagement with, or denial of, threats. In other words, self‐affirmations are speculated to reduce implications of individual threats on self‐integrity and allow individuals to orient themselves in an open and even‐handed manner towards threat management (Sherman & Cohen, 2006). Support has been found for these arguments across various studies. For example, self‐affirmations have been shown to increase individuals' acceptance of health‐risk information, intentions to follow health behaviour recommendations, and health behaviour after receiving health‐threatening information (e.g., Epton et al., 2015; Harris et al., 2007; Havranek et al., 2012; Ogedegbe et al., 2012; van Koningsbruggen et al., 2009). Despite an abundance of evidence pointing towards the effects of self‐affirmations on reducing defensive responses, little work has been undertaken on the relationships between self‐affirmation and the broad categories of approach and avoidance coping. Avoidance coping involves movement away from a stressor, filtering out of information, or turning away from threatening cues (Anshel et al., 2010). Approach coping, on the other hand, involves attempts to deal with a stressor by actively approaching and addressing it, such as by planning and attempting to solve the problems associated with it (Finset et al., 2002). Relative to avoidance coping, approach coping is often considered as a more adaptive orientation for long‐term wellbeing (e.g., Herman‐Stahl et al., 1995; Taylor & Stanton, 2007). Importantly for the present work, the orientations and behaviours often elicited by self‐affirmations—such as active coping and the sourcing of informational support—are aligned with the broad category of approach coping (see e.g., Eisenberg et al., 2012), whereas the defensive orientations that self‐affirmations are purported to reduce (e.g., denial, disengagement) are aligned with aspects of avoidant coping (see e.g., Carver, 1997).

Much of the research on the benefits of self‐affirmations have involved experimentally‐induced self‐affirmations, highlighting the value of prompts to self‐affirm on individuals' wellbeing, especially in the short‐term (see Howell, 2017, for review). Relatively little research has been undertaken on spontaneous self‐affirmations—self‐affirmations that are unprompted and that occur in response to naturalistic self‐threats. Of the limited work on spontaneous self‐affirmations, most studies highlight an array of positive psychological and cognitive outcomes these types of affirmations produce (Brady et al., 2016; Emanuel et al., 2018; Ferrer et al., 2015; Harris et al., 2019; Taber, Howell, et al., 2016; Taber, Klein, et al., 2016). For example, using cross‐sectional data from a large, nationally representative adult sample from the USA, Emanuel et al. (2018) found that spontaneous self‐affirmations were aligned to greater happiness, hopefulness, optimism, subjective health, and personal self‐efficacy, and less anger and sadness. With respect to coping, Harris et al. (2019) discovered that spontaneous self‐affirmations were positively related to dispositional coping strategies that were reflective of approach orientations, such as planning and positive reappraisal, and negatively related to strategies that were reflective of avoidant orientations, such as self‐blame. Despite recent research attention on spontaneous self‐affirmations, more work is needed to highlight the outcomes of naturally‐occurring self‐affirmations, and research across time (rather than cross‐sectional in nature) is particularly encouraged (Emanuel et al., 2018).

The current study incorporates a repeated assessment design involving participants' completion of up to seven surveys across 21 days. The purpose of the study is to determine relationships between spontaneous self‐affirmations and broad coping style and wellbeing (aspects of hedonic and eudaimonic). The repeated assessment design permits interpretations relating to inter‐individual (between‐person) and intra‐individual (within‐person, across time) variability. Significant insights have been provided for a variety of health‐related phenomena using these designs (e.g., Dimmock et al., 2021; Krause et al., 2020; Rebar et al., 2018). Because self‐affirmations are demonstrated to make people less defensive and more open, even‐handed, and able to approach threats rationally (Epton et al., 2015; Sherman & Cohen, 2006), it is hypothesized that spontaneous self‐affirmations will be positively associated with approach coping at both within‐ and between‐person levels, and negatively associated with avoidance coping at both within‐ and between‐person levels. It is also hypothesized that spontaneous self‐affirmations will be positively related to positive affect and a composite measure of eudaimonic well‐being, and negatively related to negative affect, at both between‐ and within‐subjects levels. Analyses will incorporate relevant covariates; for example, given that threat intensity may bear an expression on self‐affirmation use, coping style, and wellbeing (e.g., Carver et al., 1989; Folkman & Lazarus, 1980; Fry & Prentice‐Dunn, 2005; Harris & Napper, 2005; Napper et al., 2014), threat intensity will be included as a covariate in the analyses. In addition, ‘outcome’ variables were used as covariates in analyses in which they were not assessed as outcomes. More details on our inclusion of covariates are provided in the sections below.

## 2 METHOD

### 2.1 Participants

Because one of the primary aims of the study was to investigate associations in within‐person deviations, only data from participants who provided at least 2 assessments were included in the study. Of the 165 students who completed a survey, 109 completed more than one, so were included in the study analyses. Comparisons were made between those who were included versus excluded on the basis of completing more than one survey. These comparisons revealed no significant differences in age (*t [10.02]* = 0.52*, p* = 0.62), gender (χ^2^ [3] = 2.61, *p* = 0.46), self‐affirmations (*t [66.62]* = 1.56*, p* = 0.12), threat intensity (*t [66.12]* = 0.22, *p* = 0.82), eudaimonic wellbeing (*t [67.74]* = −0.62*, p* = 0.54), positive affect (*t [65.07]* = 1.38*, p* = 0.17), or negative affect (*t [73.84]* = 1.53*, p* = 0.13), but those excluded had higher approach coping (*t [64.40]* = 4.06*, p* < 0.01; *M Δ* = 0.38) and higher avoidance coping (*t [68.41]* = 3.22*, p* < 0.01; *M Δ* = 0.26) than those whose data were retained.

Of the 109 participants, most were female (71.6%; 26.6% male; 1.8% non‐binary or preferred not to report). Participants were aged 17–60 years (*M* = 22.26, *Mdn* = 19, *SD* = 8.16). Students were recruited using a university participation scheme and received course credit for their involvement in the study. The James Cook University Human Research Ethics Committee granted ethics approval (Approval number: H8106). Sufficient power was reached for medium and large effects for between‐person hypothesis testing, with power of 1 – *β* = 0.99 for large between‐person effects (*f* ^2^ = 0.35), and power of 1 – *β* = 0.78 for medium between‐person effects (*f* ^2^ = 0.15; Faul et al., 2007). The *R* package simr (Green & MacLeod, 2016) was used to estimate the power for the within‐person effects. The study was powered at 80% (95% confidence interval [CI]: 44.39–97.48) for small within‐person effects and 99% for medium or large effects (95% CI: 69.15–100.00). Given the seven repeated assessments and 109 participants, power for within‐person effects for multilevel modelling was also met for small‐medium and large effects (Amatya & Bhaumik, 2018).

### 2.2 Procedure

Recruitment of participants occurred during the calendar years of 2020 and 2021. Participants were asked to complete a set of seven online surveys, with the provision of each survey separated by a period of three days. Email reminders were sent to participants each three days to encourage continued engagement with the surveys. Each survey was embedded within Qualtrics software (Qualtrics, Provo, UT), and participants were encouraged to undertake the surveys at a time and location suitable for themselves. Aside from questions on demographic information (age, gender), which were included in the first survey only, all surveys included the same questionnaire items. All surveys invited participants to detail a “single issue or event that occurred within the last three days that initially caused at least some stress”. To avoid confusion among participants, phrasing in relation to self‐threats, self‐worth, or self‐integrity was avoided. Participants' accounts of stressors were screened at the conclusion of data collection to ensure they contained implications for self‐integrity.

### 2.3 Measures

In addition to obtaining details about individual stressors, each questionnaire contained items relating to intensity of the threat created by the stressor, coping strategies employed in response to the stressor, self‐affirmations used in response to the stressor, and wellbeing. After describing an individual issue or event that initially caused stress in the previous three days, participants were instructed to consider their immediate reaction to the stressor. Specifically, participants were invited to answer the following two items according to their immediate response before they were able to employ a coping strategy: “How stressful was the event or issue?” and “How unpleasant was the event or issue?”. These items have previously been incorporated into a validated event appraisal measure by Dunkley and colleagues (e.g., Dunkley, 2003; Dunkley et al., 2014). Our measure excluded one item on threat duration that is often used by Dunkley and colleagues; this item was deemed to be problematic for use in our study due to possible overriding effects from coping, and has been shown to have the weakest factor loading on global threat appraisal (see Dunkley, 2003). Each question was answered on a 5‐point Likert scale with responses ranging from 1 (not at all) to 5 (extremely). The scores were averaged to produce a single threat‐intensity score between 1 and 5, with lower scores indicating lower levels of threat intensity. Inter‐item reliability for scores derived from the threat intensity measure was strong (*ρ* = 0.90).

To measure coping strategies, Carver's (1997) Brief Coping Orientation to Problems Experienced (COPE) questionnaire was used. A situation‐specific adaptation of the questionnaire (tense of the verbs were changed) was utilized to allow assessment of situational coping in response to participants' specific stressors. The Brief COPE has been designed to assess coping in studies with multiple outcome measures to reduce burden on participants. Although the Brief COPE measures 14 types of coping, researchers often employ factor analysis on the scale and utilize more parsimonious structures (see Solberg, Gridley, & Peters, 2021). In our study, varimax principal component factor analysis was conducted to test which items loaded onto subscales (*KMO‐Criterion = 0.74; Bartlett χ* ^*2*^ *(378) = 7825.61, p* *<* *0.01).* The cumulative proportion of variability explained by two factors was 51.8%, with no statistically significant additional variability gained with additional factors. The subscales were created based on factor loadings of >0.40 onto a single scale and were interpretable as approach and avoidance scales. The substance use, humour, religion, and acceptance subscales in the original Brief COPE were omitted on the basis of this factor analysis. The 10‐item approach subscale included items such as “In response to this particular stressful situation, I…concentrated my efforts on doing something about the situation,” and “…got emotional support from others,” and the 10‐item avoidance subscale included items such as "…turned to work or other activities to take my mind off things,” or “…gave up trying to deal with it.” Each question was answered on a four‐point Likert scale anchored at 0 (*I haven't been doing this at all*) and 3 (*I've been doing this quite a lot*). Scores for each subscale were averaged to create a single score each for approach and avoidant coping (Carver, 1997). Internal consistency estimates (*α*) for scores derived from the measures in this study were 0.82 for approach and 0.81 for avoidant coping.

A validated 13‐item measure of spontaneous self‐affirmations was developed by Harris et al. (2019). However, with a goal of limiting survey length at each time point, we measured spontaneous self‐affirmations with a two‐item scale based on a previously used two‐item measure (e.g., Emanuel et al., 2018; Ferrer et al., 2015; Taber, Howell, et al., 2016; Taber, Klein, et al., 2016). When other researchers have used this two‐item scale, participants have often been asked to rate the extent to which they think about “values” and “strengths” when feeling threatened or anxious. To aid interpretation and understanding for our sample (participants were young adults—most of them were first year university students), and to capture a broad range of spontaneous self‐affirmations (e.g., related to social relationships; see Harris et al., 2019), we made a small modification to each item. Specifically, we asked participants to report on the extent to which they thought about “what is important to you in life” and “positive qualities or strengths” when feeling threatened or anxious. Responses were recorded on a 4‐point Likert scale anchored at 0 (*I didn't do this at all*) and 3 (*I did this a lot*), with higher scores indicating greater use of self‐affirmation in response to the particular stressor. Scores on the two items had good reliability as indicated by the Spearman‐Brown coefficient (*ρ* = 0.88). If participants scored above 0 across these two items, they were asked to describe their self‐affirmations in an open‐ended paragraph. Two researchers familiar with self‐affirmation theory verified that the written responses represented self‐affirming thoughts in domains separate to the domains detailed as stressful in the earlier section of the survey. The written responses were found to represent self‐affirmations across broad areas reflecting goals, relationships, values, roles, and attributes/characteristics.

Eudaimonic wellbeing was measured using a seven‐item questionnaire (*α* = 0.87) previously used by Sanchez and Garcia (2009), which asked participants to rate how they generally felt since they last took the survey. The measure addressed autonomy (e.g., “I felt that I had a say in what happened”), relatedness (e.g., “I felt that people cared about me”), and self‐esteem (e.g., “I was satisfied with myself”). Items were scored on a 4‐point Likert type scale anchored at 1 (*strongly disagree*) and 4 (*strongly agree*). The seven items were averaged to create a single eudaimonic wellbeing score between 1 and 4, with lower scores indicating lower eudaimonic wellbeing (*α* = 0.85). This instrument was chosen as a measure of eudaimonic wellbeing given its previous use in assessments of daily fluctuations in life satisfaction (Sanchez & Garcia, 2009).

Positive and negative affect were measured using the International Positive and Negative Affect Scale Short Form (I‐PANAS‐SF), which contains 10 items across positive and negative subscales (Thompson, 2007; Watson et al., 1988). Items were scored on a 5‐point Likert scale anchored at 1 (*strongly disagree*) and 5 (*strongly agree*). Each positive and negative subscale was averaged to create a single score between 1 and 5, with lower scores indicating lower levels of positive (*α* = 0.77) or negative (*α* = 0.82) affect. Thompson (2007) has found the I‐PANAS‐SF to be a reliable, valid, and efficient means of measuring positive and negative affect.

### 2.4 Data management & analyses

Intraclass correlations (ICCs) were used to evaluate degree of change in threat intensity, self‐affirmations, approach coping, avoidance coping, positive affect, negative affect, and eudaimonic wellbeing. The hypotheses were tested using multilevel modelling to account for within‐person nesting of data over the seven time points in the *lme4* (Bates et al., 2015; Bauer et al., 2006; Krull & MacKinnon, 2001) package of *R* version 3.6.2 (R Core Team, 2019). Maximum likelihood estimation was used to account for missingness. In separate models, approach coping, avoidance coping, eudaimonic wellbeing, positive affect, and negative affect were regressed onto between‐ and within‐person variables of self‐affirmation, allowing for the interpretation of self‐affirmation as a predictor of each outcome variable. Threat intensity was included as a covariate in all models to control for the influence of threat intensity on coping and wellbeing. Additionally, to test the independent effects of self‐affirmation on coping strategies and wellbeing outcomes, avoidance coping was included as a covariate in the model predicting approach coping, approach coping was included as a covariate in the model predicting avoidance coping, positive and negative affect were included as covariates in the model predicting eudaimonic wellbeing, eudaimonic and negative affect were included in the model predicting positive affect, and eudaimonic wellbeing and positive affect were included in the model predicting negative affect.

Between‐person effects were tested with variables calculated as each individual's mean score across all occasions (referred to throughout as *Overall* scores). Within‐person effects were tested with variables calculated as deviations from each individual's overall score per occasion (referred to throughout as *Occasion‐Specific* scores). Prior to model estimation, univariate and multivariate data quality checks for normality and outliers of the study variables used in analyses were conducted. Additionally, throughout analyses, model assumption testing was conducted for linearity of relationships, normality of residuals, and homoscedasticity throughout analyses. It was confirmed that there were no assumptions violated. To estimate effect sizes, Pseudo‐R^2^ was calculated based on marginal variance estimates (Johnson, 2014; Nakagawa et al., 2017; Nakagawa & Schielzeth, 2013). These effect sizes are only interpretable using variability of the between‐ and within‐person levels, so they provide comparisons of the amount of variability explained in the outcome of self‐affirmation, but not of overall self‐affirmation compared to occasion‐specific self‐affirmation.

## 3 RESULTS

### 3.1 Sample characteristics

In total, there were 540 assessments from 109 participants, with 50% of the sample responding to five or more surveys. There were 64 cases of missing response items (<0.1%). Study variable descriptive statistics are shown in Table 1. The ICCs revealed that threat intensity varied the most over time, with only about one‐third of variability accounted for at the between‐person level. All other study variables had ICCs close to, or greater than, 0.50, indicating that half or more of the variability was accounted for by individual differences rather than change over time. The intercorrelations of study variables reveal medium positive associations of self‐affirmation with approach coping, eudaimonic wellbeing, and positive affect. Additionally, threat intensity had medium positive associations with avoidance coping and negative affect, and a medium negative association with eudaimonic wellbeing. Approach coping had a medium positive association with positive affect, whereas avoidance coping had a medium negative association with eudaimonic wellbeing and a positive medium association with negative affect. Eudiamonic wellbeing showed a medium positive association with positive affect and a medium negative association with negative affect.

**TABLE 1:** Descriptive statistics, intraclass correlations, and inter‐correlations

Variable	Mean (SD)	Range	ICC	2.	3.	4.	5.	6.	7.
1. Self‐Affirmation	1.36 (0.93)	0–3	0.52	−0.08	0.43	−0.11	0.34	0.42	−0.19
2. Threat intensity	3.42 (0.93)	1–5	0.31	‐‐	0.09	0.45	−0.30	−0.15	0.47
3. Approach coping	1.38 (0.60)	0–3	0.48		‐‐	0.03	0.23	0.44	0.00
4. Avoidance coping	0.91 (0.60)	0–3	0.64			‐‐	−0.55	−0.24	0.57
5. Eudaimonic wellbeing	2.92 (0.61)	1–4	0.67				‐‐	0.51	−0.60
6. Positive affect	3.11 (0.77)	1–5	0.62					‐‐	−0.16
7. Negative affect	2.64 (0.91)	1–5	0.67						‐‐

*Note*: ICC: Intraclass Correlation; Correlations do not account for within‐person nesting so statistical significance is not reported.

### 3.2 Self‐affirmation and coping

The model testing the between‐ and within‐person associations of self‐affirmation with coping is shown in Table 2. Approach coping was positively associated with self‐affirmation at both the between‐ and within‐person levels, demonstrating that people who spontaneously self‐affirmed tended to employ approach coping strategies, and that on occasions when spontaneous self‐affirmation use was particularly high, so was the use of approach coping strategies. Threat intensity was positively associated with approach coping, but there was no significant association between avoidance coping and approach coping. The pseudo‐R^2^ revealed that self‐affirmation explained 20.1%, threat intensity explained 1.3%, and avoidance coping explained <1% of variability in approach coping.

**TABLE 2:** Multilevel model regression estimates for testing between‐ and within‐person associations of self‐affirmation with coping, controlling for threat intensity and other coping variable

Dependent variable	Approach coping		Avoidance coping	
	*b*	95% Confidence interval [LL, UL]	*b*	95% Confidence interval [LL, UL]
Intercept	0.64 (0.12)*	0.41, 0.87	0.29 (0.12)*	0.06, 0.52
Overall use of self‐affirmations	0.35 (0.05)*	0.25, 0.45	−0.03 (0.06)	−0.14, 0.10
Occasion‐specific use of self‐affirmations	0.18 (0.03)*	0.12, 0.24	−0.01 (0.03)	−0.06, 0.04
Threat intensity	0.09 (0.03)*	0.03, 0.14	0.21 (0.02)*	0.17, 0.24
Approach coping	‐‐	‐‐	−0.04 (0.04)	−0.11, 0.03
Avoidance coping	−0.03 (0.05)	−0.13, 0.07	‐‐	‐‐

*Note*: 540 observations from *N* = 109, *p < 0.05. LL = Lower Limit; UL = Upper Limit.

No significant relationships—at either the between‐ or within‐person levels—were observed between self‐affirmation use and avoidance coping, although higher threat intensity was associated with more use of avoidance coping (pseudo‐R^2^ of self‐affirmation and approach coping <1.0%; pseudo‐R^2^ of threat intensity = 10.8%).

### 3.3 Self‐affirmation and wellbeing

The results of the models testing the between‐ and within‐person associations of self‐affirmation and wellbeing are shown in Table 3. Eudaimonic wellbeing was positively associated with self‐affirmation at the between‐person but not within‐person level, such that people who used more self‐affirmation tended to have more positive eudaimonic wellbeing than those who used less self‐affirmation overall, but eudaimonic wellbeing was not impacted by within‐person deviations in self‐affirmation. Threat intensity was not significantly associated with eudaimonic wellbeing. There was a significant positive association of eudaimonic wellbeing with positive affect and a negative association with negative affect. The pseudo‐R^2^ calculations revealed that 7.2% of variability in eudaimonic wellbeing was explained by self‐affirmation, <1.0% of variability was explained by threat intensity, 6.2% of variability was explained by positive affect, and 20.1% of variability was explained by negative affect.

**TABLE 3:** Multilevel model regression estimates for testing between‐ and within‐person associations of self‐affirmation with aspects of wellbeing, controlling for threat intensity and other wellbeing variables

Dependent variable	Eudaimonic wellbeing		Positive affect		Negative affect	
	*b*	95% Confidence interval [LL, UL]	*b*	95% Confidence interval [LL, UL]	*b*	95% Confidence interval [LL, UL]
Intercept	2.96 (0.13)*	2.70, 3.22	1.55 (0.26)*	1.01, 2.08	3.70 (0.23)*	3.25, 4.15
Overall use of self‐affirmations	0.17 (0.04)*	0.08, 0.25	0.33 (0.07)*	0.20, 0.46	0.00 (0.08)	−0.15, 0.15
Occasion‐specific use of self‐affirmations	0.02 (0.02)	−0.03, 0.06	0.11 (0.03)*	0.04, 0.18	−0.03 (0.03)	−0.10, 0.03
Threat intensity	−0.02 (0.02)	−0.06, 0.02	−0.05 (0.03)	−0.10, 0.01	0.22 (0.03)*	0.17, 0.27
Eudaimonic wellbeing	‐‐	‐‐	0.41 (0.06)*	0.29, 0.53	−0.63 (0.06)*	−0.75, −0.51
Positive affect	0.19 (0.03)*	0.14, 0.25	‐‐	‐‐	0.01 (0.04)	−0.07, 0.10
Negative affect	−0.29 (0.03)*	−0.344, −0.24	0.03 (0.04)	−0.06, 0.12	‐‐	‐‐

*Note*: 540 observations from *N* = 109, *p < 0.05. LL = Lower Limit; UL = Upper Limit.

Self‐affirmation use at both the between‐person and within‐person level was associated with positive affect, in that people who used more self‐affirmation reported more positive affect overall, and when people used more self‐affirmations than usual, they tended to have more positive affect than was typical for them. Threat intensity was not significantly associated with positive affect. There was a positive, significant association of eudaimonic wellbeing with positive affect, but no significant association between positive and negative affect. Between‐person self‐affirmation explained 3.9% of variability in positive affect and within‐person self‐affirmation explained another 1.1% of variability. Less than 1.0% of variability in positive affect was explained by each of threat intensity, eudaimonic wellbeing, and negative affect.

Negative affect was not significantly associated with self‐affirmation use at either the between‐person or within‐person level. More threat was associated with more negative affect, eudaimonic wellbeing was negatively associated with negative affect, and positive affect was not associated with negative affect. Self‐affirmation and positive affect explained <1.0% of variability, threat intensity explained 1.8% of variability, and eudaimonic wellbeing explained 6.0% of negative affect.

## 4 DISCUSSION

The aim of this study was to test associations between spontaneous self‐affirmations, coping style, and wellbeing. Consistent with hypotheses, results indicated that spontaneous self‐affirmations were associated with approach coping at both between‐ and within‐person levels, but unexpectedly, spontaneous self‐affirmations were not associated with avoidance coping at either within‐ or between‐person levels. In other words, individuals who typically used more spontaneous self‐affirmations also tended to utilize more approach (but not less avoidance) coping, and on occasions when individuals employed more than usual spontaneous self‐affirmations, they also used more approach (but not less avoidance) coping. Most of our expectations relating to spontaneous self‐affirmations, eudaimonic wellbeing, and positive affect were supported; spontaneous self‐affirmations were associated with positive affect at both between‐ and within‐person levels, and with eudaimonic wellbeing at the between‐person level. Unexpectedly, our hypothesis that spontaneous self‐affirmations would be inversely related to negative affect at both between‐ and within‐person levels was not supported, with no significance observed at either level.

It is proposed in self‐affirmation theory that self‐affirmations attenuate defensive responses to self‐threats, reducing orientations to dismiss, avoid, and/or deny self‐threatening information (Sherman & Cohen, 2006; Steele, 1988). Previous research has supported this premise, indicating that self‐affirmations facilitate a calibrated and rational stance to self‐threats (see Epton et al., 2015). Our study adds partial support to these arguments, highlighting positive associations—at both between‐ and within‐person levels—between spontaneous self‐affirmations and a composite approach coping orientation. Only partial support was provided for the arguments on the basis that we expected negative—but observed no significant—associations between spontaneous self‐affirmations and avoidance coping. Causality between variables cannot be inferred, but regardless, the observed associations between spontaneous self‐affirmations and general coping orientations support the premise that self‐affirmations are aligned with orientations to address—rather than avoid—self‐threats.

Results involving spontaneous self‐affirmations and wellbeing also supported a limited body of literature in which self‐affirmations have been shown to improve both hedonic and eudaimonic wellbeing (e.g., Nelson et al., 2014). Individuals who, in general, used more spontaneous self‐affirmations reported higher scores on our composite measure of eudaimonic wellbeing, which contained items relating to autonomy, relatedness, and self‐esteem. As a caveat, however, our expectations relating to *negative affect* were not supported. The finding that spontaneous self‐affirmations were related to positive affect but not (negatively related to) negative affect is interesting, and reasons for the existence of these relationships is deserving of future scrutiny.

Important to the interpretation of results in the present study is the inclusion of various covariates in analyses. In particular, we controlled for both threat intensity and other outcome variables in analyses, allowing for a stronger test of independent prediction from self‐affirmation. With respect to threat intensity, not only is it possible that spontaneous self‐affirmations are more likely to be adopted in the face of significant, relative to minor, threats, but it is also possible that self‐affirmations bear a stronger relationship with approach coping and wellbeing in the face of substantial threats. To the extent that significant threats have more potential to undermine wellbeing, the employment of self‐affirmations and a concomitant approach coping orientation has more potential to attenuate negative affect in response to these threats. Our study was used to investigate unique associations between self‐affirmations, coping, and wellbeing—using threat intensity as a covariate—so further work is needed to investigate the role of threat intensity in moderating the use and effects of spontaneous self‐affirmations.

Aside from the results outlined above in relation to hypothesis testing, it is notable that participants' mean score on self‐affirmation was 1.36 (on a 0–3 scale), indicating that self‐affirmations were frequently adopted in response to everyday stressors. Our data are encouraging to the extent that the long‐term value of self‐affirmations relies on their adoption and utility *outside of* controlled laboratory environments. Our sample of undergraduate psychology students does not represent the broader population, but our research supports a small body of work indicating that spontaneous self‐affirmations are frequently adopted in response to naturally‐occurring threats (e.g., Emanuel et al., 2018; Taber, Howell, et al., 2016).

### 4.1 Limitations, future directions, and conclusions

Our repeated assessment design allowed participants to reflect on recent experiences pertaining to individual threats, coping, and wellbeing. The three‐day period between each survey was chosen in an effort to ensure (a) a close enough window for participants to recollect individual threats and their responses to these threats, and (b) enough duration of time for individuals to have experienced at least one threat. It was possible, however, that some participants may have experienced multiple threats in each three‐day window, and although questions in each survey were directed towards a single threat (and responses to that threat), it may have been difficult for individuals to disentangle responses across multiple threats. An alternative design in which individuals are invited to report on threats, coping, and wellbeing in the hours following exposure to self‐threatening information may reduce the possibility of contamination from multiple experiences. Further, although we achieved power at both between‐ and within‐person levels, our response rate, with only 50% of the sample completing five or more surveys, may be improved in future research. We used an email reminder system to encourage participation, but in the future it may be advantageous to use text message or smartphone app reminders.

An additional consideration in relation to the present study is that of generalizability—our sample consisted of (primarily healthy) university students, and it would be interesting to explore whether the observed relationships are similar in other populations, such as those experiencing mental health concerns. In relation to such individual differences, we acknowledge that our observed between‐person relationships may have been influenced by factors such as trait self‐esteem or dispositional optimism (see e.g., Emanuel et al., 2018; Harris et al., 2019), and further work is needed to elucidate the unique role of spontaneous self‐affirmations in coping orientations and wellbeing. In undertaking such work, researchers may wish to consider relationships involving other possible dimensions of well‐being. Our measures captured experiences that are often associated with eudaimonic wellbeing and hedonic wellbeing, although some researchers incorporate additional—or different—components to these categories of wellbeing (for review, see Huta, 2015). Finally, our self‐report method of assessing coping strategies over time may have influenced natural coping orientations (including use of self‐affirmations), as per the Hawthorne effect (see e.g., Sedgwick & Greenwood, 2015).

Despite the above‐mentioned limitations, this study offers unique insight into multi‐level relationships involving spontaneous self‐affirmations, coping orientations, and wellbeing. Controlling for the influence of threat intensity and other outcome variables, the results highlighted the co‐occurrence of spontaneous self‐affirmations, approach coping, and positive wellbeing. In general, the associations between these concepts were observed at both between‐ and within‐person levels, highlighting the robustness of the associations. Spontaneous self‐affirmations appear to be undertaken in a landscape in which individuals are inclined to adopt approach coping and yield positive wellbeing, pointing towards the adaptive benefits of such affirmations. On the basis of the present work, spontaneous self‐affirmations appear useful as a simple tool for stress management, and may be particularly useful as an adaptive precursor to approach‐focussed strategies such as information seeking and planning. Practitioners may wish to adopt a two‐staged approach in promoting spontaneous self‐affirmations. First, practitioners could provide individuals with rationales for the anticipated benefits of these self‐affirmations, some of which have been documented in the present study. Such an approach would be useful in facilitating individuals' *motivation* to self‐affirm (see Vansteenkiste et al., 2018, for benefits of rationales). Second, by providing guidance on how and when individuals may use self‐affirmations—including information on the importance of focussing affirmations on domains separate to the domain/s threatened—practitioners are likely to improve individuals' ability to self‐affirm. Notwithstanding these applied recommendations, it is important that subsequent research is undertaken on the influence of spontaneous self‐affirmations on coping and wellbeing to verify and build upon the findings in the present study.

## CONFLICT OF INTEREST

The authors declare no conflicts of interest.

## AUTHOR CONTRIBUTION

Taylor‐Jane Sharouni and Rachel G. McClymont contributed equally to all aspects of this study and share first authorship. Christopher Alcorn assisted with data collection. Amanda L. Rebar and Kwok Hong Law oversaw data analysis; Ben Jackson, Nerina Caltabiano, and James A. Dimmock assisted with study design and project management. All authors collaborated to approve the final version of the manuscript.

## Data Availability

The Ethics approval for this project stated that participants would explicitly consent to the possible re‐use of their data by the researchers, but it did not permit the sharing of the collected data.
